# hUC-MSC transplantation therapy effects on lupus-prone MRL/*lpr* mice at early disease stages

**DOI:** 10.1186/s13287-023-03432-2

**Published:** 2023-08-21

**Authors:** Fengbiao Guo, Quanren Pan, Ting Chen, Shuzhen Liao, Shangmei Li, Aifen Li, Shuxian Chen, Jiaxuan Chen, Zengzhi Xiao, Hongyong Su, Lawei Yang, Chen Yang, Hua-feng Liu, Qingjun Pan

**Affiliations:** Guangdong Provincial Key Laboratory of Autophagy and Major Chronic Non-Communicable Diseases, Affiliated Hospital of Guangdong Medical University, Zhanjiang, 524001 Guangdong China

**Keywords:** SLE, Lupus mice, hUC-MSCs, B cells, Immunosuppressive

## Abstract

**Background:**

The efficacy of human umbilical cord mesenchymal stem cell (hUC-MSC) transplantation in treating systemic lupus erythematosus (SLE) has been confirmed by small-scale clinical trials. However, these trials focused on severe or refractory SLE, while few studies focused on mild SLE. Therefore, this study focused on the therapeutic effects of hUC-MSC transplantation in early-stage or mild MRL/*lpr* lupus model mice.

**Methods:**

Commercially available hUC-MSCs were transplanted into 8-week-old MRL/*lpr* mice by tail vein injection. Flow cytometry was used to analyze B cells and their subsets in the peripheral blood. Further, plasma inflammatory factors, autoantibodies, and plasma biochemical indices were detected using protein chip technology and ELISA kits. In addition, pathological staining and immunofluorescence were performed to detect kidney injury in mice.

**Results:**

hUC-MSC transplantation did not affect the mice’s body weight, and both middle and high dose hUC-MSC transplantation (MD and HD group) actually reduced spleen weight. hUC-MSC transplantation significantly decreased the proportion of plasmablasts (PB), IgG1^−^ PB, IgG1^+^ PB, IgG1^+^ memory B (MB) cells, IgG1^+^ DN MB, and IgG1^+^ SP MB cells. The hUC-MSC transplantation had significantly reduced plasma levels of inflammatory factors, such as TNF-α, IFN-γ, IL-6, and IL-13. Pathological staining showed that the infiltration of glomerular inflammatory cells was significantly reduced and that the level of glomerular fibrosis was significantly alleviated in hUC-MSC-transplanted mice. Immunofluorescence assays showed that the deposition of IgG and IgM antibodies in the kidneys of hUC-MSC-transplanted mice was significantly lower than in the control.

**Conclusion:**

hUC-MSC transplantation could inhibit the proliferation and differentiation of peripheral blood B cells in the early-stage of MRL/*lpr* mice, thereby alleviating the plasma inflammatory environment in mice, leading to kidney injury remission. The study provides a new and feasible strategy for SLE treatment.

**Supplementary Information:**

The online version contains supplementary material available at 10.1186/s13287-023-03432-2.

## Introduction

Systemic lupus erythematosus (SLE) is an autoimmune disease characterized by the aberrant activation of B cells and autoantibody production [1]. The pathogenic autoantibodies combine with the corresponding autoantigens; they are then deposited in the skin, joints, glomeruli, and other parts of the body leading to multiple organs and system damage (e.g., lupus nephritis; LN) [2–4]. Previous epidemiological reports show that Guangdong province in southern China has high-SLE incidences [5, 6]. A combination of hormones and cyclophosphamide is the classical therapy for SLE treatment; it can effectively alleviate the progression of SLE and improve the survival and prognosis of patients [4]. However, these therapies have serious side effects, such as infection, bone marrow suppression, secondary malignancy, and disease recurrence after drug withdrawal [2]. Recently, a B cell-targeted biological agent, belimumab, which explicitly inhibits the B cell activating factor (BAFF), has been shown to alleviate clinical symptoms and reduce the production of immunological abnormalities in SLE patients. Belimumab uses a single target and has been used for moderate to severe SLE treatment [7–9]. Further, it cannot inhibit plasma cells (PC) and the conversion of memory B (MB) cells [10]. Therefore, there is an urgent need to develop novel, safer, and more effective strategies for SLE treatment.

The successful treatment of patients with early lupus without side effects, especially in preventing the occurrence of new mild SLE manifestations, thus delaying disease progression, remains a big challenge. The current treatment of early mild lupus includes, but is not limited to, low-dose corticosteroids, antimalarial drugs (antimalarials), vitamin D, and statins [11]. However, although these drugs seem effective in treating early-stage SLE, some SLE manifestations cannot be controlled entirely. In other words, these drugs may not be sufficient in blocking disease progression and even progression to end-stage renal disease (ESRD). In addition, the side effects of long-term use of these drugs, such as hydroxychloroquine (HCQ), have gained widespread concern, as HCQ is ineffective in preventing the occurrence of severe SLE manifestations [12]. Therefore, alternative or additional strategies are required to successfully treat early lupus [12].

Mesenchymal stem cell (MSC) transplantation has recently emerged as a promising therapy for patients with SLE owing to MSCs’ strong immunosuppressive function [13]. Many studies have reported that MSC transplantation is safe and effective in patients with advanced (severe) or refractory SLE [14–18]. In addition, compared with bone marrow-derived MSCs (BM-MSCs), human umbilical cord MSCs (hUC-MSCs) show lower immunogenicity and more vigorous proliferation/differentiation ability [19–21]. To date, 18 clinical studies worldwide on the use of MSCs in the treatment of SLE have been recorded on the website of *Clinical Trial* (https://clinicaltrials.gov/). Almost all these clinical trials have been conducted or will be conducted on patients with advanced (severe) or refractory SLE.

Based on the effective treatment of MSC transplantation in advanced (severe) or refractory SLE patients, MSC transplantation should be a promising therapy in the early stages of lupus. Therefore, this study aimed to investigate the therapeutic effects of hUC-MSC transplantation at an early disease stage in lupus-prone MRL/*lpr* mice, which will provide a novel strategy for treating early SLE.

## Materials and methods

### Animal studies

Five MRL/MPJ and twenty MRL/*lpr* mice (female, age 3–4 weeks, 13–15 g) were purchased from Shanghai Slac Laboratory Animal Company [license no. SCXK (Hu) 2017–0005]. Mice were housed in a specific pathogen-free barrier facility which was characterized by a 12 h light/dark cycle, 22–25 °C, and 40–60% humidity with free access to water and forage. All the animal studies were approved by the laboratory animal ethical committee (LAEC) of Guangdong Medical University (license no. GDY2103031). Animal welfare was reviewed during the application of the animal experiments by the LAEC of Guangdong Medical University and was monitored during the experiments by the employees of the LAEC of Guangdong Medical University. To euthanatize mice before collecting tissue and blood samples, mice were intraperitoneal injection of sodium pentobarbital (100 mg/kg). After that, cervical dislocation was performed after mice were unresponsive to toe pinch.

### Mesenchymal stem cells (MSCs)

Five to seven generations of human umbilical cord mesenchymal stem cells (hUC-MSCs) were obtained from Hunan Yuanpin Biotech Co. Ltd. (Changsha, China). The hUC-MSCs used in this study showed fibroblast-like morphology and plastic adherence. Further, they met the criteria set by the International Society for Cellular Therapy (ISCT) to define MSCs [22].

### Mesenchymal stem cell (MSC) transplantation

**Fig. 1:**
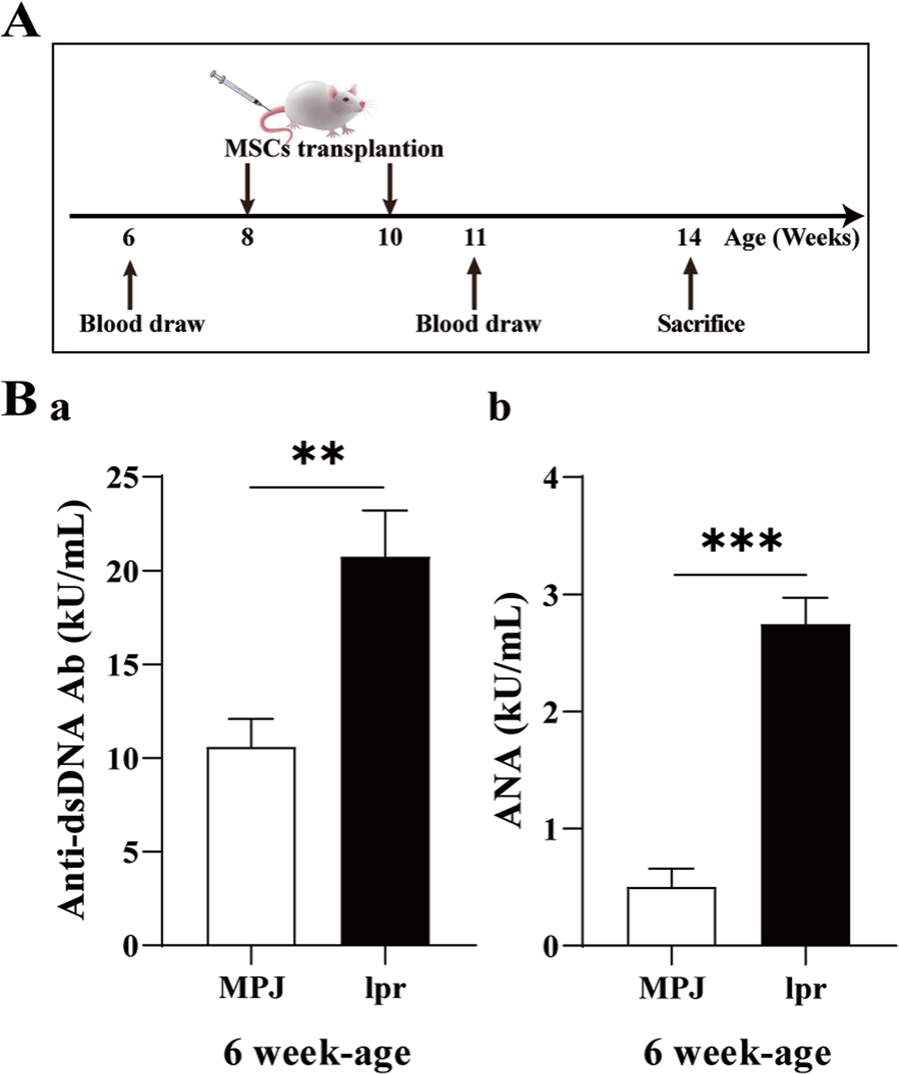
Alternation of autoantibody levels in MRL/*lpr* mice at early stage of disease. **A.** Procedure timeline. **B.** Plasma levels of anti-ds DNA antibodies (**a**) and Antinuclear Antibodies (ANA) (**b**) in 6-week-old MRL/*lpr* mice showed a significant increase compared to the MRL/MPJ mice. **P < 0.01; ***P < 0.001. The number of mice is *n *= 5

Female MRL/*lpr* mice were divided into four groups by random table method (five mice in each group, *n* = 5): control (Ctrl), low-dose hUC-MSC transplantation (LD), middle-dose hUC-MSC transplantation (MD), and high-dose hUC-MSC transplantation (HD). hUC-MSC was suspended in 300 μl saline and transplanted twice (Ctrl: saline, LD: 0.4 × 10^6^ cells/10 g each injection, MD: 0.8 × 10^6^ cells/10 g each injection, HD: 1.2 × 10^6^ cells/10 g each injection) via the tail vein at the 8th and 10th week. All mice were sacrificed in the 14th week for further analysis (Fig. 1A).

### Urine protein measurement

In metabolic cages, urine samples were collected by keeping mice overnight (12 h). After centrifugation at 3000 rpm and 4 °C for 10 min, the supernatant was immediately stored at − 80 °C. Samples were thawed on ice before the urine protein was assessed. Proteinuria concentration and the urinary albumin/creatinine ratio (ACR) were determined using an autoanalyzer (Cobas 8000, Roche, Switzerland). Then, the total 24-h urinary protein was calculated based on the concentration, volume, and duration of urine collection.

### Blood sample collection and processing

Blood sample collection was performed by cardiac puncture after anesthesia at 14 weeks old. The blood samples were collected with EDTA anticoagulant tube and centrifuged at 3000 rpm for 10 min to obtain the plasma, the precipitation is blood cells for flow cytometry (FCM) analysis, and the supernatant is plasma which was immediately stored at − 80 °C.

### Flow cytometry (FCM)

After centrifugation of mice's peripheral blood, red blood cells were lysed using a lysis buffer (BD PharmLyse Lysis Buffer; BD Biosciences, San Diego, CA, USA) to obtain a single-cell suspension. The single-cell suspension was incubated with Purified anti-mouse CD16/32 Antibody (Catalog No. 158002, BioLegend, San Diego, CA, USA) to block nonspecific antibody binding. Subsequently, a cocktail of fluorescence-conjugated antibodies, including CD19, CD138, and PD-L2, was added for staining. Following staining, the cells were fixed with 4% PFA and analyzed using the FACS Celesta™ Flow Cytometer (BD Biosciences, San Diego, CA, USA). The analysis of B cell subsets and the gate scheme in flow cytometry were performed according to previous reports (Fig. 3A) [23, 24]. Furthermore, to evaluate cell surface PD-L1 expression, hUC-MSCs were stained with Alexa Fluor 647-conjugated PD-L1 antibody (Additional file 1: Fig. S1A). All flow cytometry data were analyzed using FlowJo 10.8.1 software (FlowJo, Ashland, OR, USA). For mice lymphocyte-derived single cells, the samples were down-sampled to 30,000 cells per sample. The detailed list of fluorescence-conjugated antibodies used for flow cytometry can be found in Additional file 2: Table S1.

### Enzyme-linked immunosorbent assay (ELISA)

Plasma levels of Antinuclear Antibodies (ANA), anti-dsDNA antibodies, and complement 3 (C3) were measured using a Mouse ANA Total Ig ELISA Kit (Catalog No. 5210, Alpha Diagnostic, San Antonio, Texas, USA), a Mouse anti-dsDNA Antibodies Total Ig ELISA Kit (Catalog No. 5110, Alpha Diagnostic, San Antonio, Texas, USA), and a Mouse C3 ELISA Kit (Catalog No. 6270, Alpha Diagnostic, San Antonio, Texas, USA), respectively, according to the manufacturer’s instructions.

### Determination of plasma cytokine levels

Plasma levels of inflammatory cytokines, including interferon gamma (IFN-γ), tumor necrosis factor-alpha (TNF-α), interleukin (IL)-1β, IL-2, IL-4, IL-6, IL-10, and IL-13, were assayed using a Milliplex^®^ MAP kit (Millipore, Billerica, MA, USA), according to the manufacturer’s recommendations. Plasma levels of TGF-β1 were assayed using a Mouse TGF-β1 ELISA kit (Catalog No. VAL611, Novus Biologicals, USA) according to the manufacturer’s instructions.

### Determination of biochemical parameters

Plasma biochemical parameters, including albumin/globulin (A/G) ratio, glycocholic acid (CG), triglyceride (TG), and total bile acid (TBA), were measured using an autoanalyzer (Cobas 8000, Roche, Switzerland).

### Spleen index measurement

The MRL/*lpr* mice (14 weeks old) fasted for 12 h, and the total body weight was measured before sacrifice. Next, the spleens were dissected and weighed. The spleen index is presented as spleen/body weight.

### Pathology assessment of kidneys

Kidneys were harvested at the time of sacrifice and fixed with 4% paraformaldehyde in PBS. The kidneys were then embedded in paraffin and sectioned (1.5 μm). Sections were stained with hematoxylin and eosin (H&E), periodic acid-Schiff (PAS), and Masson’s trichrome stain, following a blind assessment by an experienced pathologist. The quantification of renal histopathological changes was performed as previously described in reference [23, 24]. The scores assigned to mesangial proliferation and PAS^+^ deposition ranged from 0 to 4, indicating the severity (0: absent; 1: mild; 2: mild-moderate; 3: moderate; 4: severe). Similarly, the grades for crescents, tubular lesions (atrophy, casts, dilatation, inflammatory infiltrates), and vasculitis were each scored on a scale of 0–4, representing the extent of their presence in the kidney sections. The maximum score for each mouse was 20, with 4 points allocated to mesangial hyperplasia, PAS^+^ deposits, crescents, tubulopathy, and vasculitis, respectively.

### Immunofluorescence

Immunofluorescence staining was performed on 3 µm paraffin-embedded sections of the kidneys and spleens from 14-week-old lupus mice. The sections underwent dewaxing and hydration steps, followed by heat-mediated antigen retrieval using either sodium citrate (10 mM, pH 6; Catalog No. P0083, Beyotime, Shanghai, China) or Tris–EDTA (10 mM Tris, 1 mM EDTA, pH 9; Catalog No. C1038, Solarbio, Beijing, China, G1480) via microwave for 20 min. To block nonspecific antigens, 5% bovine serum albumin (BSA) was used. Primary antibodies were incubated overnight at 4 °C, while fluorescent secondary antibodies were incubated for 1 h at room temperature. A diluted DAPI solution (1:3 dilution; Catalog No. C1005, Beyotime, Shanghai, China) was applied for 10 min to stain the cell nuclei. After washing with PBS, the sections were sealed using anti-fluorescence quenching sealed tablets. The primary antibodies, fluorescent primary antibodies, and secondary antibodies used for immunofluorescence staining can be found in Additional file 2: Table S1. Immunofluorescence images of the kidneys were acquired using a laser scanning confocal microscope (Leica TCS SP5) and analyzed with Leica LAX software (Leica, Wetzlar, Germany). Immunofluorescence images of spleen T cells and B cells were obtained using an Olympus VS200 fluorescence microscope and analyzed with OlympusVIA software (Olympus, Japan).

### Statistical analyses

All data were statistically analyzed using GraphPad Prism 8.0.2. The Kolmogorov–Smirnov and Shapiro–Wilk tests were used to determine whether the data conformed to a normal distribution. For the comparison of normally distributed data between the two groups, the general t test was used when the variance was uniform, and the Welch t test was used when the variance was uneven. One-way ANOVA was used to compare the mean values of more than two groups of normally distributed data. Brown-Forsythe and Welch ANOVA tests were used to compare the mean values of more than two groups of non-normally distributed data. In addition, the Mann–Whitney U test was used for comparisons between two variables, and the Kruskal–Wallis test was used to compare multiple variables. *P* < 0.05 was considered statistically significant in all statistical methods.

## Results

### Alternation of autoantibody levels in MRL/*lpr* mice at early stage of disease.

To investigate the disease status of MRL/*lpr* mice before hUC-MSCs transplantation, plasma anti-double-stranded DNA (anti-ds DNA) antibody and anti-nuclear antibody (ANA) levels of 6-week-old mice were detected by ELISA kit, with MRL/MPJ as the normal control, as shown in Fig. 1B. The results showed that the plasma levels of anti-ds DNA (a) antibody and ANA (b) in 6-week-old MRL/*lpr* lupus model mice were significantly higher than those of MRL/MPJ mice. This indicates abnormal levels of autoantibodies (anti-ds DNA antibodies and ANA) in the early-stage of lupus mice (i.e., 6 weeks of age), and this is consistent with previous reports [25].

### Effects of hUC-MSC transplantation on autoantibodies and spleen weight of mice.

**Fig. 2:**
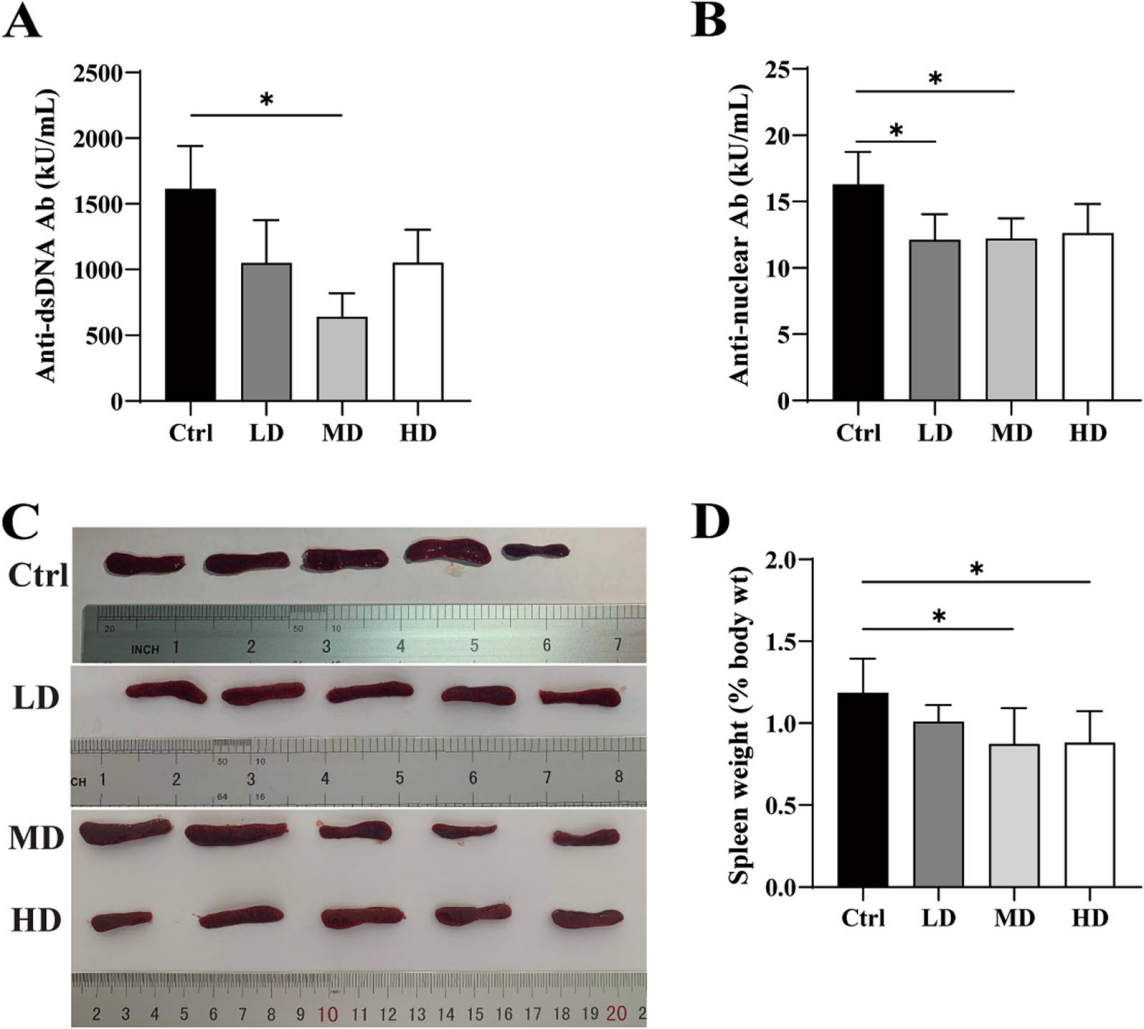
hUC-MSC transplantation effects on autoantibodies and spleen weight of 14-week-old MRL/*lpr* mice. **A** hUC-MSC transplantation significantly decreases the plasma levels of anti-ds DNA antibodies (**a**) and Antinuclear Antibodies (ANA) (**b**). **B** Images of spleens.** C** Spleen weight ratio of mice in the MSC transplantation groups (MD and HD groups) showed a decrease compared with the Ctrl. (Ctrl: control, LD: low dose, MD: middle dose, HD: high dose). The number of mice is *n *= 5

To explore the therapeutic effect of hUC-MSCs transplantation on early-stage of lupus mice, ELISA was used to detect plasma antibody related indexes of MRL/*lpr* mice, and the results were shown in Fig. 2. The results showed that the hUC-MSCs transplantation at middle dose (MD group) could reduce the level of anti-ds DNA antibody (Fig. 2A), and the hUC-MSCs transplantation at low dose (LD group) and MD group could significantly reduce the level of ANA (Fig. 2B). Mice were sacrificed at 14 weeks of age, and spleen weight ratio of mice was monitored. Spleen images were shown in Fig. 2C and Additional file 3: Fig. S2. Spleen weight of mice in the middle- and high-dose hUC-MSCs transplantation group (MD group and HD group) was significantly lower than that of the control group (Fig. 2D). These results suggest that hUC-MSCs transplantation in early-stage of disease has an inhibitory effect on spleen enlargement, which may be related to the inhibition of lymphocyte proliferation by hUC-MSCs.

### hUC-MSC transplantation affected the differentiation of peripheral blood B cell subsets in mice

**Fig. 3:**
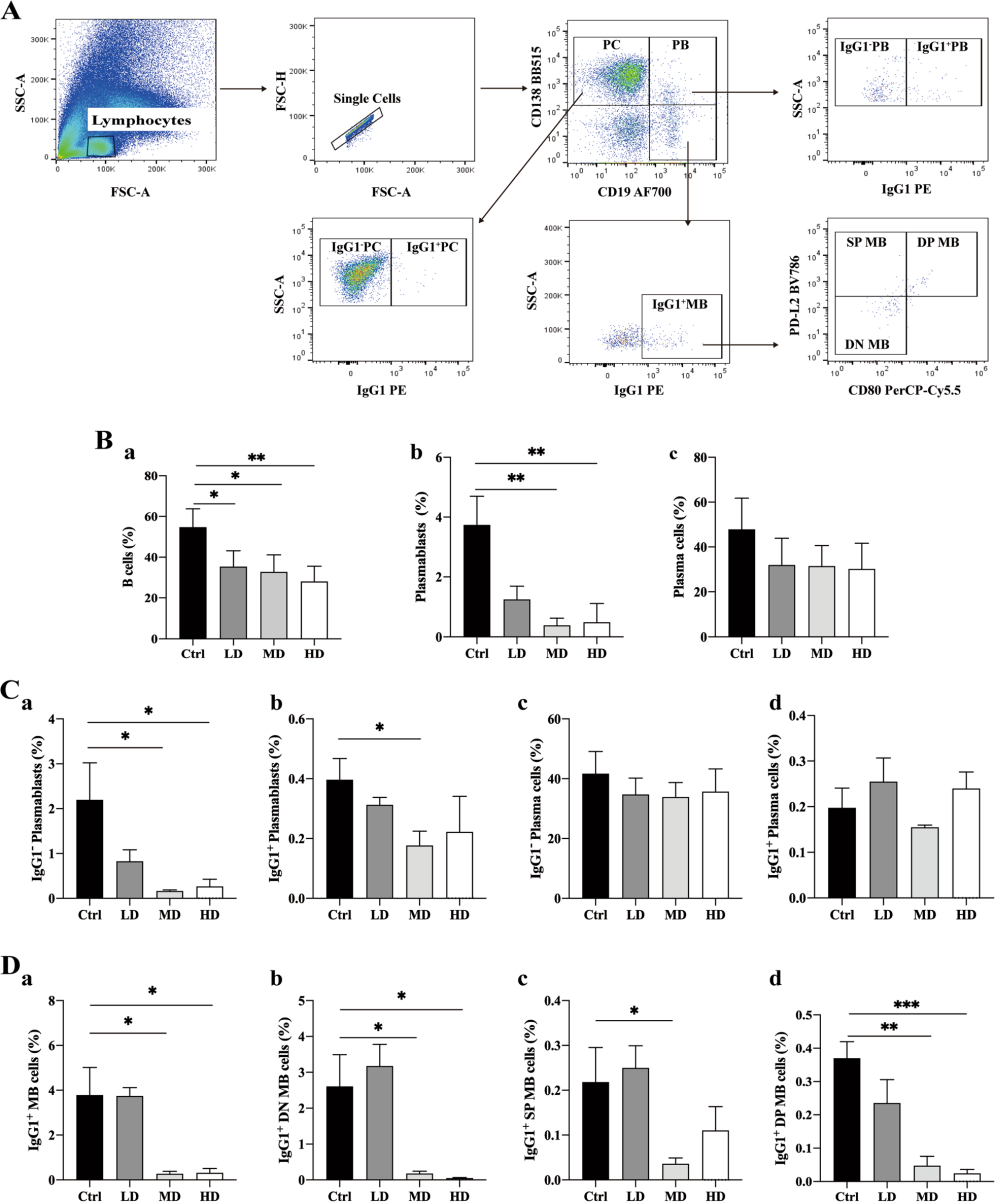
hUC-MSC inhibition of B cells subtype in 14-week-old MRL/*lpr* mice. **A** The peripheral blood flow cytometry (FCM) gating strategy in MRL/*lpr* mice.** B** Proportion of total B cells (**a**), plasma cells (PC) (**b**), and plasmablasts cell (PB) (**c**) in lymphocytes. **C** The proportion of IgG1^−^ PB (**a**), IgG1^+^ PB (**b**), IgG1^−^ PC (**c**), and IgG1^+^ PC (**d**) in lymphocytes. **D** The proportion of total IgG1^+^ MB cells (**a**), double-negative (DN) MB cells (**b**), single-positive (SP) MB cells (**c**), and double-positive (DP) MB cells (**d**) in total B cells. *P < 0.05; **P < 0.01. (Ctrl: control, LD: low dose, MD: middle dose, HD: high dose). The number of mice is *n *= 5

It was found that hUC-MSCs transplantation reduced the spleen weight ratio in early lupus mice, which may be related to reduced lymphocyte proliferation. The results showed that the levels of plasma autoantibodies (ANA and anti-ds DNA antibodies) were significantly increased in the early-stage of lupus disease, and the alternation of B-cell subsets after hUC-MSCs transplantation was further detected. Surface or intracellular labeled antibodies of different B cell subsets were used for labeling, and the changes of each B cell subset were analyzed by flow cytometry, as shown in Fig. 3. Lymphocytes were first gated and then single-cell gated using FSC-A and FSC-H diagrams. After the single-cell gate, the total number of plasma cells (PC, CD19^−^CD138^+^ cells) and plasmablasts cells (PB, CD19^+^CD138^+^ cells) can be identified in the CD19 and CD138 maps. Secondly, IgG1^+^ plasma cells, IgG1^−^ plasma cells, IgG1^+^ plasmablasts cells, and IgG1^+^ plasmablasts cells can be identified using IgG1. In addition, CD19^+^CD138^−^IgG1^+^ B cells could be identified as total IgG1^+^ memory B cells (MB). Three subsets of total IgG1^+^ memory B cells were further identified by CD80 and PD-L2. They were double-positive memory B cells (DP MB, CD80^+^PD-L2^+^), single-positive memory B cells (SP MB, CD80^−^PD-L2^+^), and double-negative memory B cells (DN MB, CD80^−^PD-L2^−^) (Fig. 3A).

The data showed that hUC-MSCs transplantation could significantly decrease the total number of B cells (including the total number of plasma cells and plasmablasts, CD19^+^CD138^−^B cells) and plasmablasts (PB), but no effected on the total number of plasma cells (PC) (Fig. 3B). Further analysis of B cell subsets showed that the number of IgG^−^ PB cells (a) and IgG1^+^ PB cells (b) in the MD and HD groups was significantly lower than that in the Ctrl group (Fig. 3C). However, hUC-MSCs transplantation had no effect on IgG1^−^ PC cells (c) and IgG1^+^ P cells (d) in peripheral blood of lupus mice.

In addition, the number of memory B-cell subsets was measured by flow cytometry, as shown in Fig. 3D. The number of total IgG^+^ memory B (MB) cells was significantly decreased in the MD and HD groups compared with the Ctrl group (a). Further B cell subsets analysis showed that hUC-MSCs transplantation significantly reduced the proportion of DN MB (b), SP MB (c), and DP MB (d). These results indicate that hUC-MSCs can regulate the proliferation and differentiation of B cells and affect the production of inflammatory cytokines and antibodies, thus alleviating disease progression in lupus mice, but the mechanism is unclear.

### hUC-MSC transplantation affected the differentiation of spleen T cells and B cell subsets in mice

**Fig. 4:**
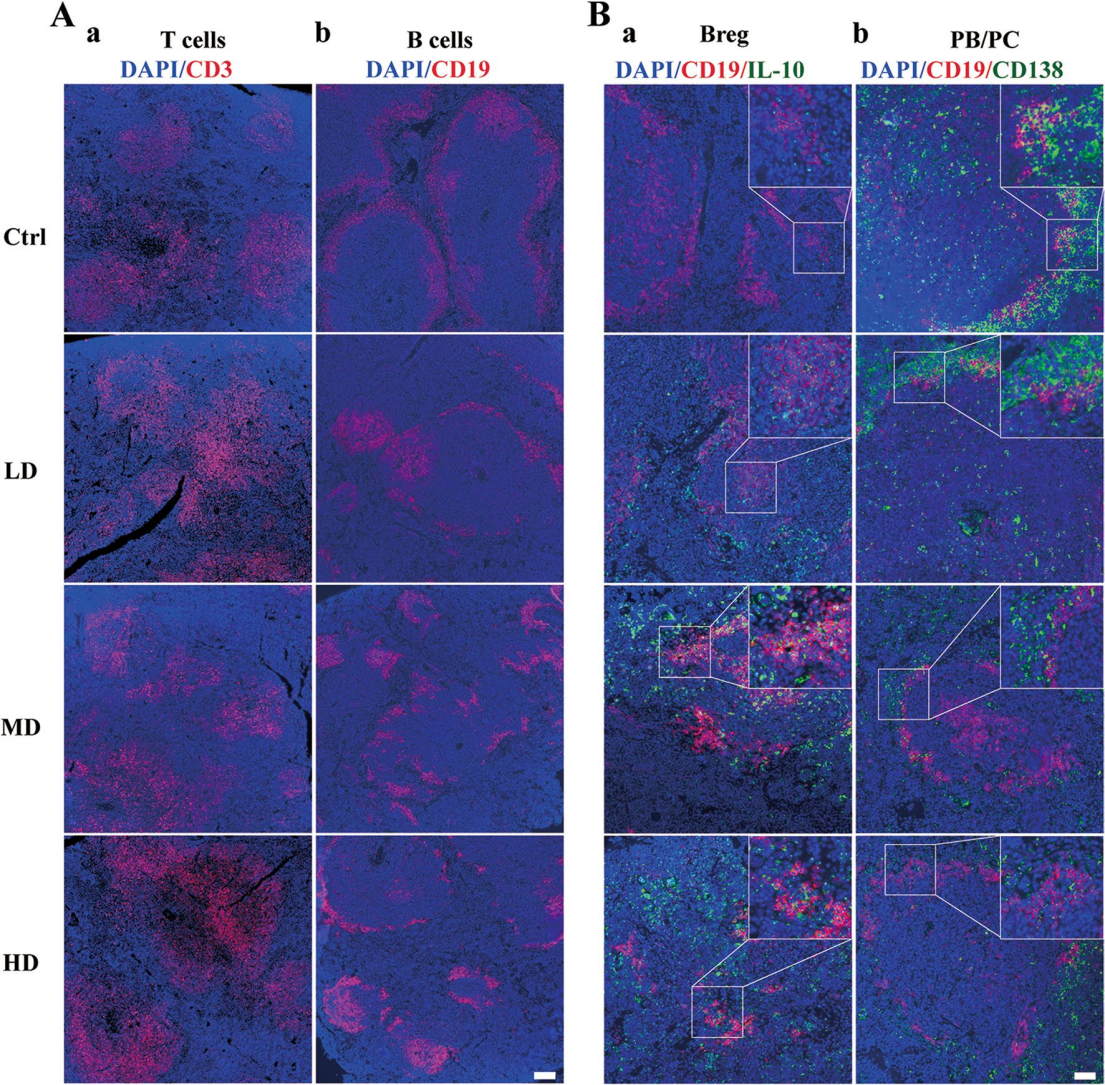
Effects of hUC-MSC transplantation on spleen T cells and B cells in 14-week-old MRL/*lpr* mice. **A** Representative images of total T cells (**a**) and total B cells (**b**) in the spleens of each group of mice. Blue fluorescence represents DAPI, red fluorescence represents CD3 or CD19, and the scale bar is 100 μm. **B** Representative images of B cell subsets in the spleens of mice from each group, including Breg cells (**a**), plasmablasts (PB), and plasma cells (PC) (**b**). The images in the upper right corner are magnified five times. Blue fluorescence represents DAPI, red fluorescence represents CD19, and green fluorescence represents IL-10 or CD138, with a scale bar of 50 μm. The number of mice is *n *= 5

Investigation into the distribution of T cell and B cell subsets in the spleen of lupus mice following hUC-MSCs treatment was carried out using immunofluorescence, as depicted in Fig. 4 and Additional file 1: Fig. S1B. Although there were no significant differences in the proportions of total T cells and total B cells in the spleen between the hUC-MSCs treatment group and the Ctrl group (Fig. 4A), notable alterations were observed within the B cell subpopulation. Compared to the Ctrl group, the proportion of Breg cells with immunosuppressive effects (yellow fluorescence) increased in the spleen of hUC-MSCs-treated mice, with the MD group exhibiting the most significant increase (Fig. 4B). Furthermore, in the MD group, there was a remarkable reduction in the proportions of plasma cells (green fluorescence) and plasmablasts (yellow fluorescence) associated with autoantibody production after hUC-MSCs treatment (Fig. 4B). Similarly, the proportions of Tfh cells, Th1 cells, Th2 cells, and Th17 cells, which are related to the pathogenesis of lupus, exhibited varying degrees of decrease within the T cell subsets of the spleen in mice treated with hUC-MSCs compared to the Ctrl group (Additional file 1: Fig. S1B). On the contrary, compared to the Ctrl group, mice in the MD group exhibited a discernible inclination toward an augmented population of Treg cells in the spleen. These results suggest that early administration of hUC-MSCs can ameliorate the distribution of T cell and B cell subsets in the spleen of lupus mice, increase the proportion of Breg and Treg cells, and reduce the proportions of plasma cells, plasma blast cells, Tfh cells, Th1 cells, Th2 cells, and Th17 cells. hUC-MSCs may exert their effects by modulating the distribution of T cell and B cell subsets, thereby potentially slowing the progression of lupus disease.

### hUC-MSC transplantation decreased the plasma levels of inflammatory cytokines

**Fig. 5:**
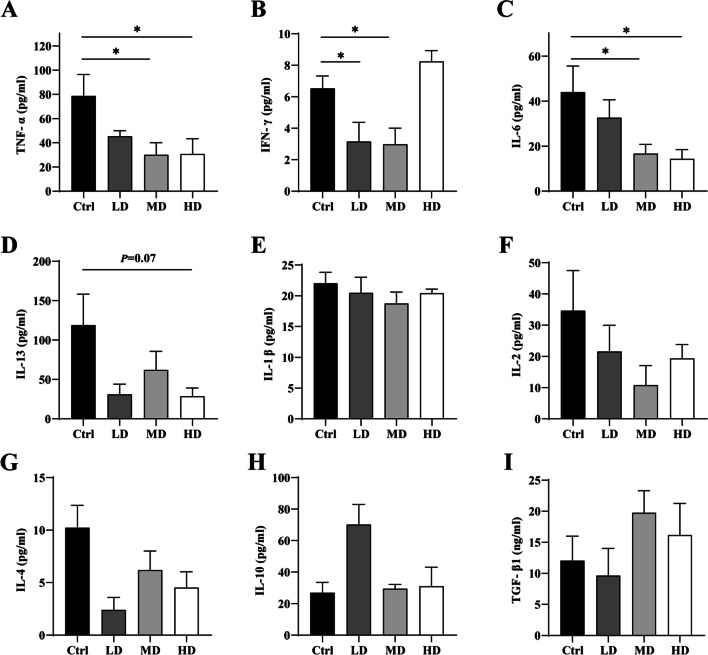
hUC-MSC transplantation effects on plasma levels of inflammatory cytokines in 14-week-old MRL/*lpr* mice. hUC-MSC transplantation significantly decrease the plasma levels of TNF-α (**A**), IFN-γ (**B**), IL-6 (**C**), and IL-13 (**D**), but hUC-MSCs transplantation had no effect on the levels of IL-1β (**E**), IL-2 (**F**), IL-4 (**G**), IL-10 (**H**), and TGF-β1 (**I**). *P < 0.05. (Ctrl: control, LD: low dose, MD: middle dose, HD: high dose). The number of mice is *n *= 5

Plasma samples were tested using protein panel technology and ELISA. The results showed that the levels of plasma inflammatory factors TNF-α (Fig. 5A), IFN-γ (Fig. 5B), and IL-6 (Fig. 5C) in MD group were significantly lower than those in Ctrl group, and the levels of TNF-α (Fig. 5A), IL-6 (Fig. 5C) and IL13 (Fig. 5D) in HD group were significantly lower than those in Ctrl group. The level of IFN-γ (Fig. 5B) in the LD group was also significantly lower than that in the Ctrl group. However, hUC-MSCs transplantation had no effect on the levels of IL-1β (Fig. 5E), IL-2 (Fig. 5F), IL-4 (Fig. 5G), IL-10 (Fig. 5H), and TGF-β1 (Fig. 5I). The results showed that hUC-MSCs transplantation could relieve the plasma inflammatory environment and reduce the plasma inflammatory cytokine levels in lupus mice.

### hUC-MSC transplantation alleviated renal function

**Fig. 6:**
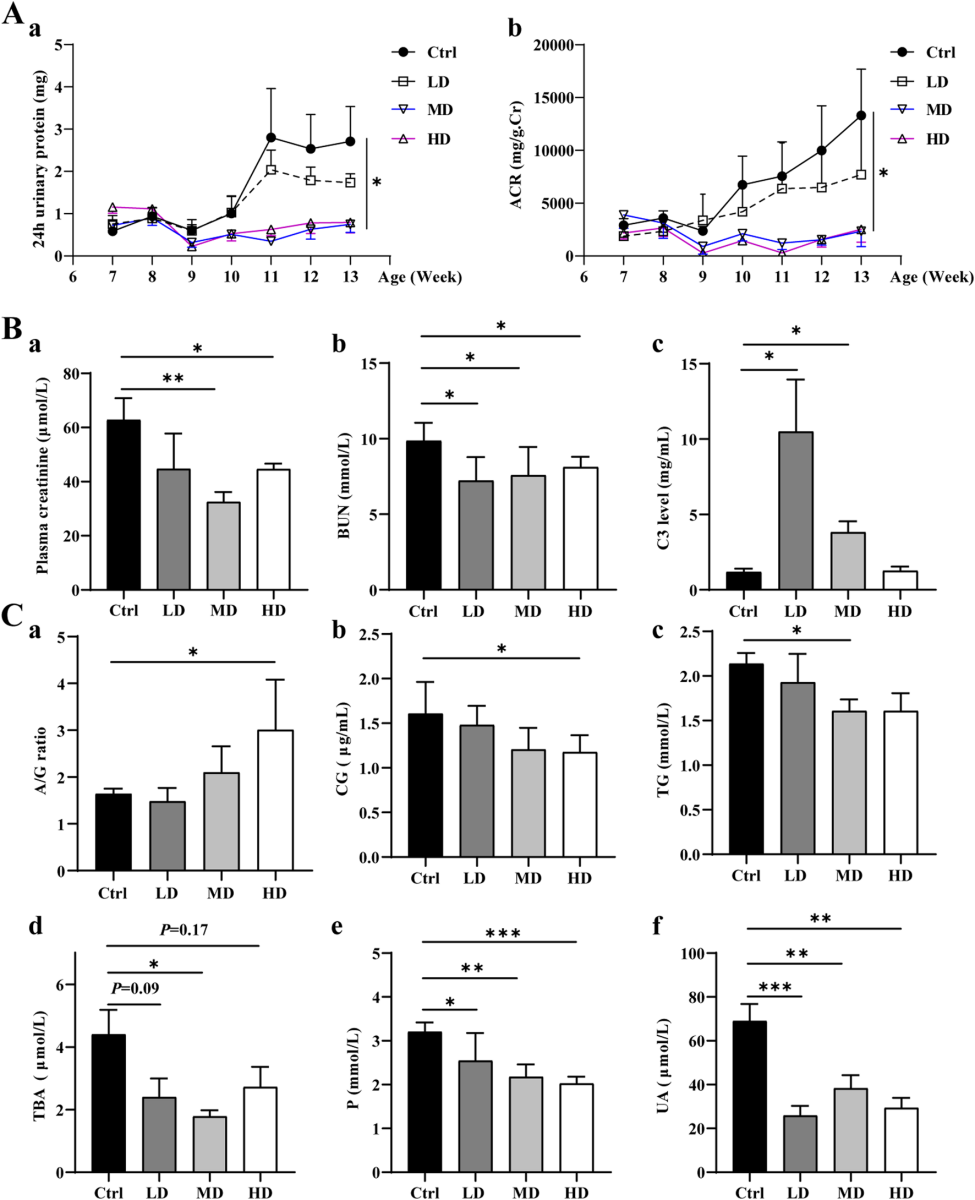
hUC-MSC transplantation alleviated renal function in 14-week-old MRL/*lpr* mice. **A** The 24 h urinary protein (**a**) and albumin/creatinine ratio (ACR) (**b**) in the middle-dose hUC-MSC transplantation (MD) showed a significant decrease compared to the control (Ctrl). **B** The plasma levels of creatinine (**a**) and blood urea nitrogen (BUN) (**b**) in the MD and HD groups were significantly decreased compared to the Ctrl, and the plasma levels of complement 3 (C3) (**c**) in LD and MD groups was significantly increased compared to the Ctrl. **C** The plasma level of albumin/globulin (A/G) (**a**) and glycocholic acid (CG) (**b**) in the high-dose hUC-MSC transplantation (HD) group increased compared to the Ctrl, the plasma levels of triglyceride (TG) (**c**) and total bile acid (TBA) (**d**) in the MD group showed a reduction, and hUC-MSC transplantation significantly decreased the plasma levels of inorganic phosphorus (P) (**e**) and Uric Acid (UA) (**f**). *P < 0.05; **P < 0.01, ***P < 0.001. (Ctrl: control, LD: low dose, MD: middle dose, HD: high dose). The number of mice is *n *= 5

Since lupus disease is very likely to involve the kidneys, renal function is assessed by measuring urinary protein levels and other relevant indicators, as shown in Fig. 6. 24-h urinary protein (a) and albumin/creatinine ratio (ACR) (b) of MD and HD groups were significantly lower than those of the Ctrl (Fig. 6A). Compared with the control group, plasma creatinine (a) and blood urea nitrogen (BUN) (b) levels in the hUC-MSCs transplantation group were significantly decreased, and plasma complement C3 (c) levels in the LD and MD groups were higher than those in the Ctrl group (Fig. 6B). In addition, the results of plasma biochemical indexes of lupus mice were shown in Fig. 6C, and the A/G ratio (a) of the HD group were higher than those of the Ctrl group. The plasma levels of glycocholic acid (CG) (b) and triglyceride (TG) (c) levels in the HD or MD group were significantly lower than those in the Ctrl group, respectively. Plasma levels of total bile acid (TBA) (d), inorganic phosphorus (P) (e) and Uric Acid (UA) (f) in hUC-MSCs transplantation group were significantly lower than those in Ctrl group. Those results showed that hUC-MSCs transplantation could improve kidney function and plasma biochemical indexes of lupus mice.

### hUC-MSCs transplantation can improve kidney injury

**Fig. 7:**
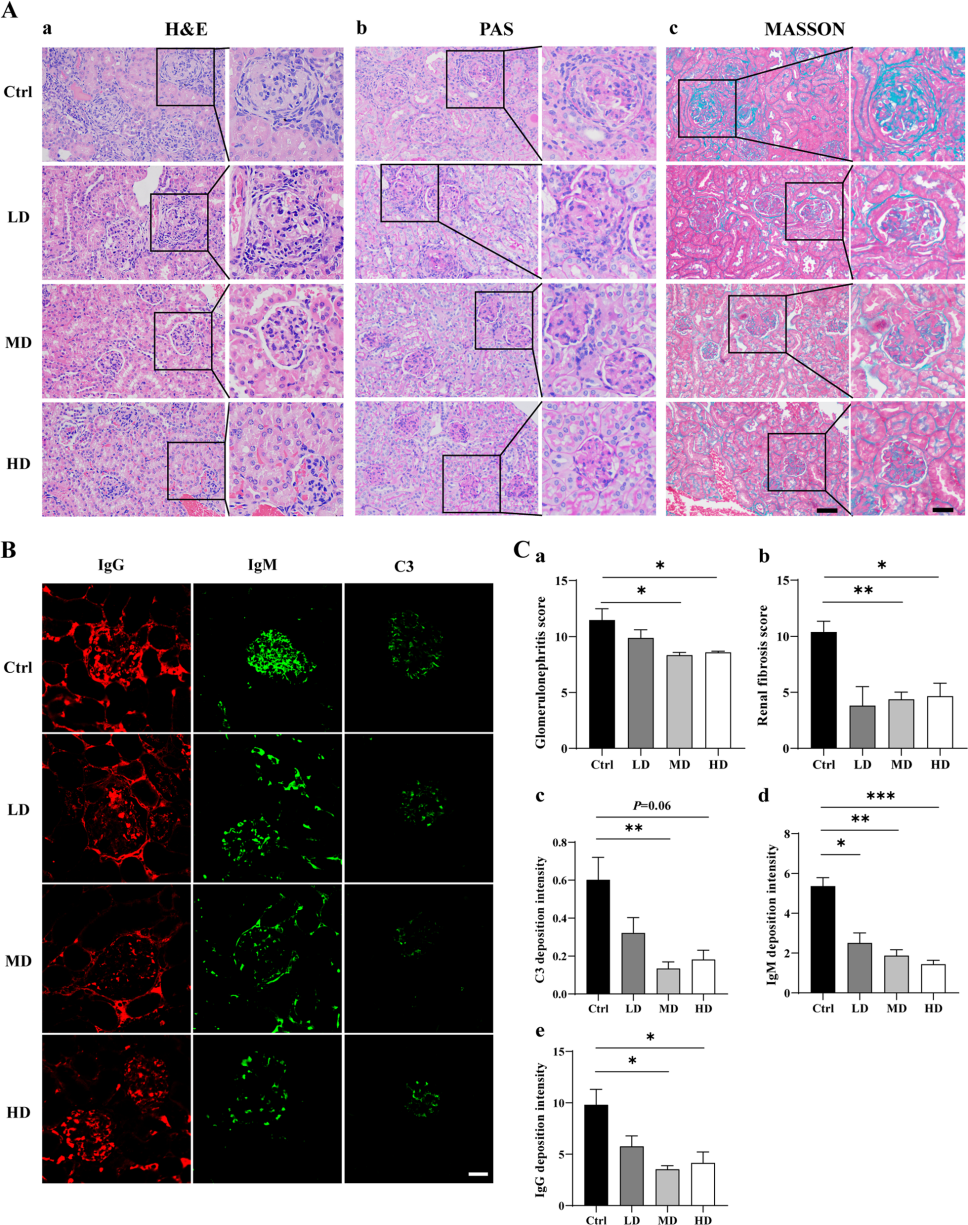
hUC-MSC transplantation improved renal damage in 14-week-old MRL/*lpr* mice. **A** Pathological staining. Glomerular inflammatory cell infiltration significantly reduced, and glomerular fibrosis significantly improved in MSC transplanted mice compared to the Ctrl. Scale bar: 50 μm and 25 μm, respectively. **B** Immunofluorescence assay. IgG and IgM antibody deposition in the kidneys of MSC transplanted mice were significantly lower than in the Ctrl group. Scale bar: 25 μm. **C** Statistical diagram of H&E staining (**a**), MASSON staining (**b**), C3 immunofluorescence staining (**c**), IgM immunofluorescence staining (**d**), and IgG immunofluorescence staining (**e**). (Ctrl: control, LD: low dose, MD: middle dose, HD: high dose). The number of mice is *n *= 5

In addition to the detection of kidney function related indicators, MRL/*lpr* mice kidneys were collected and made into paraffin wax. H&E, PAS, and Masson pathological staining were used to observe and analyze the protective effect of MSCs transplantation on the kidneys of MRL/*lpr* mice, and the results were shown in Fig. 7. Pathological staining results showed that the glomerular inflammatory cell infiltration and glomerular fibrosis of hUC-MSCs transplanted mice were significantly reduced (Fig. 7A). Immunofluorescence results showed that the deposition of C3, IgM, and IgG antibodies in the kidneys of MRL/*lpr* mice transplanted with hUC-MSCs was significantly lower than that of the control group (Fig. 7B). Statistical diagram of pathological staining and immunofluorescence staining as shown in Fig. 7C (a. H&E staining, b. MASSON staining, c. C3 immunofluorescence staining, d. IgM immunofluorescence staining, e. IgG immunofluorescence staining). These results suggest that hUC-MSCs transplantation can alleviate kidney injury in early lupus mice.

## Discussion

MSCs could be isolated and cultured from bone marrow and adult tissues such as the placenta, umbilical cord, umbilical cord blood, and adipose tissue. However, hUC-MSCs are the most widely used. MSCs are pluripotent, self-replicating stem cells that can differentiate into various mature specialized cells [26]. Previous studies have shown that MSCs have anti-inflammatory effects [27], regulate immune systems (adaptive and innate) [28, 29], induce immune tolerance and homing to injured tissues, have low immunogenicity, and have a low resistance to immune rejection. Therefore, MSCs can treat various diseases, including SLE [28, 30]. The effects of MSC transplantation in treating SLE animal models have been successively reported in basic research [31, 32]. Clinical studies have also reported that transplantation of MSCs derived from allogeneic bone marrow or umbilical cord can effectively treat refractory SLE [16, 18], achieving clinical remission or low lupus disease activity state (LLDAS). Unfortunately, MSC transplantation for SLE therapy has been limited to small-scale clinical trials and has not been applied in clinical transformation in recent decades, and its long-term efficacy requires further observation. In addition to the high-cost factor, there are still many issues that need to be resolved before clinical application.

In this study, we found that the plasma levels of ANA and anti-ds DNA antibodies in 6-week-old MRL/*lpr* mice were significantly higher than those in MRL/MPJ mice. These results indicate that the secretion of autoantibodies (ANA and anti-ds DNA antibodies) is abnormal in the early-stage of lupus onset (i.e., six weeks of age), suggesting that this may be related to B cell abnormalities. These results are consistent with previous studies [33, 34]; however, the mechanism of autoantibody production by B cell abnormalities is unknown.

As we know, SLE is a chronic, multisystem complex autoimmune disease characterized by abnormal activation of B cells and differentiation into memory B cells (MB), plasmablasts (PB), and plasma cells (PC). These abnormal B cells have specificity and high affinity for autoantigens and can secrete pathogenic autoantibodies, such as anti-ds DNA antibodies, Anti-phospholipid β2 glycoprotein I (β2GPI) antibodies or anti-cardiolipin antibodies (ACA), anti-RO/SSA or La/SSB antibodies, and Sm/RNP antibodies [35]. Recent studies have shown that IgG^+^ or IgG^−^ MB and PB may have different roles in the production of autoantibodies [35]. Studies have reported that some antibodies, such as Ro, La, Sm, RNP and ACA, exist for a long time in SLE patients, which may be secreted by MB and PB [36–38]. However, MB and PB do not respond to T cells or antigens and could survive for several months to decades. Immunosuppressants targeting CD20^+^ B cells (such as rituximab) are ineffective, and the therapeutic efficacy of rituximab in different SLE patients varies greatly. It may be related to the continuous secretion of autoantibodies by MB and PB which is CD20^−^. Therefore, it is suggested that the difficulty of SLE and other autoimmune diseases may be related to the continuous secretion of autoantibodies by MB and PB. MB and PB are very important for chronic disease and prognosis. In this study, the subset alteration of peripheral blood B cells was analyzed by FCM after hUC-MSC transplantation. hUC-MSC transplantation inhibited the differentiation of B cell subsets, such as total PC and PB, which decreased significantly after hUC-MSC transplantation. Further, the number of IgG^−^ PB and IgG1^+^ PB cells in the hUC-MSC transplantation groups was considerably lower than in the Ctrl. Similar results were observed for subsets of MB cells. These results indicate that hUC-MSC transplantation in the early-stage of lupus could reduce the proportion of peripheral PB and MB cells in lupus mice. This suggests that hUC-MSC transplantation may contribute to the remission of SLE disease progression by regulating B cell activation and differentiation. In other words, hUC-MSCs exert their immunosuppressive effects by regulating B cell function. Further research could be focused on isolating mouse peripheral blood B cells or co-culture them with hUC-MSCs to analyze how hUC-MSCs regulate B cell activation and differentiation, including autoantibody production.

Previous studies demonstrated that MSCs exert an immunosuppressive effect and can regulate the function of immune cells through direct contact (cell-to-cell interactions) and indirect contact (secreted cell mediators). In direct contact mode, MSCs exert immunomodulatory effects by binding their surface ligands to target cell surface receptors such as PD1-PD-L1. In the indirect contact mode, MSCs modulate the immune function of target cells by releasing exosomes or soluble factors such as transforming growth factor-β (TGF-β), indoleamine 2,3-dioxygenase (IDO), prostaglandin E2 (PGE2), galactose agglutinin-1 (Gal-1), and hepatocyte growth factor (HGF), thereby exerting immune regulatory functions [39, 40]. However, the detailed mechanisms by which MSCs exert their therapeutic effects are not yet fully understood. Based on the experimental results and previous reports, it can be hypothesized that hUC-MSCs might regulate B cell differentiation by secreting exosomes, thereby reducing the production of autoantibodies, and achieving disease remission.

Based on the B cell alternation, the plasma levels of autoantibodies and inflammatory factors were further detected using the Milliplex^®^ MAP and ELISA kits. The data showed that hUC-MSC transplantation significantly reduces the plasma levels of autoantibodies, such as anti-ANA and anti-dsDNA antibodies. But the plasma C3 levels in the LD and MD groups showed an increasing trend compared with the Ctrl. Furthermore, the levels of plasma proinflammatory factors (such as TNF-α, IFN-γ, IL-6 and IL-13) were lower in the MD and HD groups than in the Ctrl group. Surprisingly, it was also found that hUC-MSC transplantation had no effect on IL-1β, IL-2, IL-4 and IL-10. However, transplantation of high concentration MSCs (HD) transplantation could up-regulate the plasma levels of IFN-γ and decrease IL-10 levels. This may be related to many uncertain factors in high-concentration MSC transplantation. Previous studies have also reported poor therapeutic effects of high-concentration MSCs transplantation [28, 41, 42], which is consistent with our findings, but the mechanism is still unclear. In follow-up studies, we will continue to look at whether high concentrations of MSCs or multiple injections promote inflammatory responses. These results indicated that hUC-MSC transplantation in the early-stage of lupus can effectively reduce plasma inflammatory factor levels and alleviate the plasma inflammatory microenvironment.

Several studies have also reported that the clinical efficacy of MSC transplantation for treating SLE remains controversial. Another study showed that the immunoregulatory function of MSCs is not stable and has no immunosuppressive effects on lymphocytes [43, 44]. Furthermore, MSCs had no significant additional impact compared to standard immunosuppressive therapy [42]. Another study also showed that MSC transplantation did not reduce disease activity in patients with SLE, and one patient developed significant renal disease four months after the MSC transplantation [15]. Another study showed that transplantation of BALB/c mouse-derived BM-MSCs in NZB/NZW F1 lupus mice caused aggravated kidney damage, increased production of autoantibodies, such as anti-dsDNA antibodies, and increased PC ratio, leading to aggravated lupus disease [45]. The study co-cultured BM-MSCs with NZB/NZW F1 mouse PC in vitro and found that MSCs enhanced the viability and function of PC and promoted the production of IgG antibodies [45]; however, the mechanism was unclear. These results further illustrate the potential proinflammatory risk of MSCs, and the impact of this change on disease progression remains to be further verified.

On the other hand, the evaluation of renal function and renal pathology found that the 24-h urine protein content of lupus mice in the MD and HD groups were lower than the Ctrl. The results of the renal pathological staining and immunofluorescence analysis showed that the renal pathology of mice in the MD and HD groups had an improvement compared to the Ctrl; these results are similar to those of previous reports. Transplantation of BM-MSCs derived from B6 mice into NZB/NZW F1 lupus model mice did not contribute to disease progression and did not affect autoantibody production, proteinuria levels, or mortality, but did improve renal pathology and reduce lymphocytic infiltration [46]. The results demonstrate that MSC transplantation could reduce the deposition of IgG antibodies in the kidney and alleviate kidney damage to a certain extent. However, hUC-MSC transplantation may not have a repair effect on kidney damage. Still, hUC-MSC transplantation in the early-stage of the disease can reduce the risk of kidney injury secondary to SLE. Further, it has a specific preventive and protective effect on the kidney.

The reasons for the inefficacy of MSC transplantation are still unclear, and it is a critical question that impedes the clinical application of MSCs. Therefore, it is a promising strategy to maintain MSCs viability and protect against oxidative stress by preconditioning or modification in vitro, such as improvement of culture methods [47], growth factor or cytokine stimulation [48], hypoxia induction [49], and genetic modifications [50, 51] to enhance its immunosuppressive functions [52]. However, these modification strategies are still in the basic experimental stage and require more evidential support and rigorous clinical trials. Therefore, further research is being done to explore the autophagy activation of hUC-MSC transplantation in SLE treatments, as well as trying to modify MSCs from a new perspective to enhance their immunosuppressive effects and reduce proinflammatory side effects.

## Conclusion

This study comprehensively analyzed the altered frequency of peripheral B cell subsets by FCM. Further, it analyzed inflammatory cytokine plasma levels by protein chip technology and their changes associated with hUC-MSC transplantation in MRL/*lpr* mice. This study found that hUC-MSC transplantation partially alleviated disease progression and exerted a protective effect on the kidneys, the MD had the best effect, and LD and HD also had a weak effect, but some parameters in the HD group were contrary to expectations, indicating that higher concentration of hUC-MSC transplantation may cause opposite outcomes. Furthermore, the lack of a comparison with advanced lupus mice makes it impossible to determine whether hUC-MSC transplantation is better in the early stages of the disease than in the advanced stages. Therefore, further research is required on the immunotherapy effects of hUC-MSCs modification on SLE and whether hUC-MSC transplantation in the early-stage of the disease can effectively delay disease progression and maintain extended low activity.

### Supplementary Information

**Additional file 1: Figure S1.** A. MSCs have the capability to interact with B cells via the PD-1/PD-L1 pathway. The flow cytometry results demonstrated that MSCs exhibited a PD-L1 positivity rate exceeding 98% (a). In the spleens of 14-week-old MRL/*lpr* mice, CD19 and PD-1 exhibited colocalization, presenting as yellow fluorescence (b). DAPI emitted blue fluorescence, CD19 emitted red fluorescence, and PD-1 emitted green fluorescence, scale bar: 50 μm. B. Distribution of spleen T cell subsets, including Tfh cells (CXCR5-positive, green fluorescence) (a), Th1 cells (IFN-γ-positive, green fluorescence), Th2 cells (GATA3-positive, green fluorescence), Th17 cells (IL-17-positive, green fluorescence) and Treg cells (FoxP3-positive, green fluorescence). Blue fluorescence represents DAPI staining, scale bar: 50 μm. The number of mice is n=5.                               

**Additional file 2: Table S1.** Antibodies used in the study.

**Additional file 3: Figure S2.** hUC-MSC transplantation effects on spleen weight of 14-week-old MRL/*lpr* mice. Spleen weight ratio of mice in the MSC transplantation groups (MD and HD groups) showed a decrease compared with the Ctrl. (Ctrl: control, LD: low dose, MD: middle dose, HD: high dose). Compare the distance measured by two rulers of different units. The result indicated that the distance measurement was the same. The number of mice is n = 5.

## Data Availability

The data that supports the fundings of this study are available on request from the authors.

## Abbreviations

A/G ration: Albumin/globulin ratio
ACR: Albumin/creatinine ratio
ANA: Anti-nuclear antibodies
BUN: Blood urea nitrogen
CG: Glycocholic acid
Ctrl: Control
HD: High dose
hUC-MSCs: Human umbilical cord mesenchymal stem cells
IFN-γ: Interferon gamma
IL: Interleukin
LD: Low dose
MB: Memory B cells
MD: Middle dose
P: Inorganic phosphorus
PB: Plasmablasts
PC: Plasma cells
SLE: Systemic lupus erythematosus
TBA: Total bile acid
TG: Triglyceride
TGF-β1: Transforming growth factor-beta l
TNF-α: Tumor necrosis factor-alpha
UA: Uric acid
