# Supplementary material for: hUC-MSC transplantation therapy effects on lupus-prone MRL/lpr mice at early disease stages
Source: Stem Cell Res Ther. 2023 Aug 21;14:211. doi: 10.1186/s13287-023-03432-2 (PMC10441722; doi:10.1186/s13287-023-03432-2)
Supplement: Supplementary file 1 — Additional file 1: Figure S1. A. MSCs have the capability to interact with B cells via the PD-1/PD-L1 pathway. The flow cytometry results demonstrated that MSCs exhibited a PD-L1 positivity rate exceeding 98% (a). In the spleens of 14-week-old MRL/lpr mice, CD19 and PD-1 exhibited colocalization, presenting as yellow fluorescence (b). DAPI emitted blue fluorescence, CD19 emitted red fluorescence, and PD-1 emitted green fluorescence, scale bar: 50 μm. B. Distribution of spleen T cell subsets, including Tfh cells (CXCR5-positive, green fluorescence) (a), Th1 cells (IFN-γ-positive, green fluorescence), Th2 cells (GATA3-positive, green fluorescence), Th17 cells (IL-17-positive, green fluorescence) and Treg cells (FoxP3-positive, green fluorescence). Blue fluorescence represents DAPI staining, scale bar: 50 μm. The number of mice is n=5. [file 13287_2023_3432_MOESM1_ESM.docx]

**Additional file 1: Figure S1.**

Immunofluorescence analysis of T cell and B cell subsets in the spleens of lupus mice was conducted, as depicted in Figure 4 and supplementary Figure 1. Consistent with previous literature[1] [2], plasma cells and plasmablasts were identified by labeling with CD19 and CD138, while regulatory B cells (Bregs) were identified through co-staining with CD19 and IL-10 (Figure 4B). Moreover, based on previous studies[3-5], specific T cell subpopulations were characterized by utilizing CXCR5, IFN-γ, GATA3, and IL-17 as markers for T follicular helper cells (Tfh), and T helper cell (Th) subsets (Th1 cells, Th2 cells, and Th17 cells), respectively (Additional file 1: Figure S1B).

_
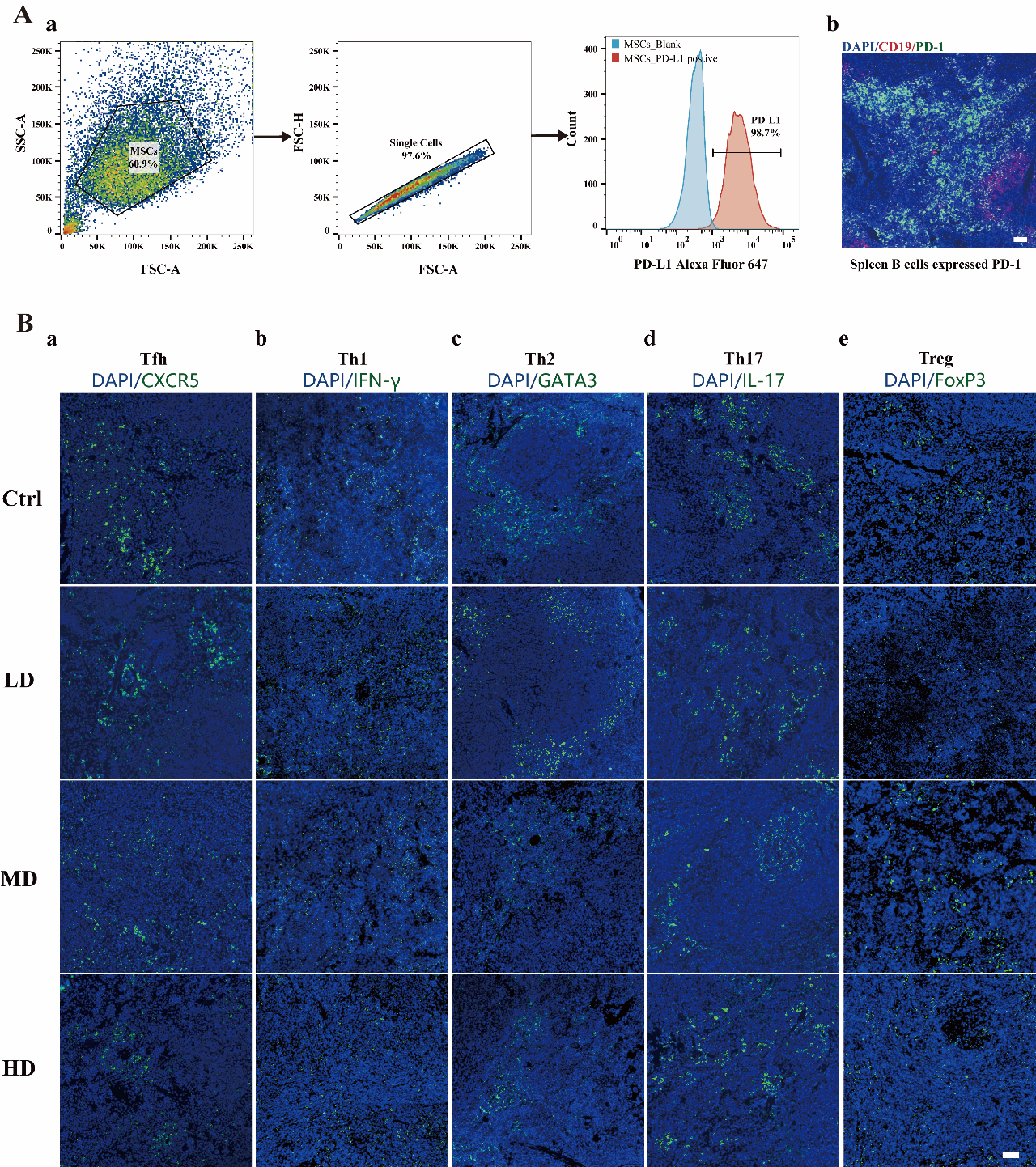
_

**Additional file 1: Figure S1. A.** MSCs have the capability to interact with B cells via the PD-1/PD-L1 pathway. The flow cytometry results demonstrated that MSCs exhibited a PD-L1 positivity rate exceeding 98% (a). In the spleens of 14-week-old MRL/*lpr* mice, CD19 and PD-1 exhibited colocalization, presenting as yellow fluorescence (b). DAPI emitted blue fluorescence, CD19 emitted red fluorescence, and PD-1 emitted green fluorescence, scale bar: 50 μm. **B.** Distribution of spleen T cell subsets, including Tfh cells (CXCR5-positive, green fluorescence) (a), Th1 cells (IFN-γ-positive, green fluorescence), Th2 cells (GATA3-positive, green fluorescence), Th17 cells (IL-17-positive, green fluorescence) and Treg cells (FoxP3-positive, green fluorescence). Blue fluorescence represents DAPI staining, scale bar: 50 μm. The number of mice is n=5.

**References**

1. Rodríguez-Lorenzo S, Konings J, van der Pol S, Kamermans A, Amor S, van Horssen J, Witte ME, Kooij G, de Vries HE. Inflammation of the choroid plexus in progressive multiple sclerosis: accumulation of granulocytes and T cells. Acta Neuropathol Commun. 2020;8:9.

2. Lin X, Wang X, Xiao F, Ma K, Liu L, Wang X, Xu D, Wang F, Shi X, Liu D, Zhao Y, Lu L. IL-10-producing regulatory B cells restrain the T follicular helper cell response in primary Sjögren's syndrome. Cell Mol Immunol. 2019;16:921-31.

3. Maneechotesuwan K, Xin Y, Ito K, Jazrawi E, Lee KY, Usmani OS, Barnes PJ, Adcock IM. Regulation of Th2 cytokine genes by p38 MAPK-mediated phosphorylation of GATA-3. J Immunol. 2007;178:2491-8.

4. Wang Y, Wang L, Yang H, Yuan W, Ren J, Bai Y. Activated Circulating T Follicular Helper Cells Are Associated with Disease Severity in Patients with Psoriasis. J Immunol Res. 2016;2016:7346030.

5. Yang C, Huang XR, Fung E, Liu HF, Lan HY. The Regulatory T-cell Transcription Factor Foxp3 Protects against Crescentic Glomerulonephritis. Sci Rep. 2017;7:1481.
