# Supplementary material for: hUC-MSC transplantation therapy effects on lupus-prone MRL/lpr mice at early disease stages
Source: Stem Cell Res Ther. 2023 Aug 21;14:211. doi: 10.1186/s13287-023-03432-2 (PMC10441722; doi:10.1186/s13287-023-03432-2)
Supplement: Supplementary file 2 — Additional file 2: Table S1. Antibodies used in the study. [file 13287_2023_3432_MOESM2_ESM.docx]

**Additional file 2: Table S1.** Antibodies used in the study.

| **Antibodies** | **Company** | **Catalog No.** | **Application** | **Dilution fold** |
| --- | --- | --- | --- | --- |
| Purified anti-mouse CD16/32 Antibody | BioLegend | 158002 | FCM | 50 |
| Alexa Fluor 700-conjugated anti-CD19 | BioLegend | 115528 | FCM | 200 |
| PE-conjugated anti-IgG1 | BioLegend | 406608 | FCM | 80 |
| BB515-conjugated anti-CD138 | BD Pharmingen | 566207 | FCM | 80 |
| Percp-cy5.5-conjugated anti-CD80 | BioLegend | 104722 | FCM | 20 |
| BV786-conjugated anti-CD273 (PD-L2) | BD Pharmingen | 741026 | FCM | 80 |
| Alexa Fluor® 647 Conjugated PD-L1 Rabbit mAb | Cell Signaling Technology | 41726S | FCM | 50 |
| PD-1 Rabbit mAb | Cell Signaling Technology | 84651S | IF | 100 |
| Goat anti-Mouse IgG, Alexa Fluor 647 | Invitrogen | A-21236 | IF | 400 |
| IgM Antibody | Novus Biologicals | NBP2-62012 | IF | 200 |
| C3 Antibody | Affinity Biosciences | DF13224 | IF | 50 |
| CD19 Monoclonal Antibody | eBioscience | 14-0194-82 | IF | 200 |
| Anti-Syndecan-1 antibody (CD138) | Abcam | ab128936 | IF | 50 |
| IL-10 Polyclonal Antibody | Bioss | bs-6761R | IF | 50 |
| Anti-CD3antibody | Abcam | ab11089 | IF | 200 |
| CXCR5 Antibody | Affinity Biosciences | DF2327 | IF | 50 |
| Interferon gamma Antibody | Affinity Biosciences | DF6045 | IF | 50 |
| GATA3 Polyclonal Antibody | Bioss | bs-1452R | IF | 50 |
| Anti-IL-17A antibody | Abcam | ab79056 | IF | 100 |
| Goat anti-Rat IgG, Alexa Fluor 647 | Invitrogen | A-21247 | IF | 200 |
| Donkey anti-Rabbit IgG, Alexa Fluor 488 | Invitrogen | A-21206 | IF | 200 |

Flow: Flow cytometry. IF: immunofluorescence.
