# Supplementary material for: hUC-MSC transplantation therapy effects on lupus-prone MRL/lpr mice at early disease stages
Source: Stem Cell Res Ther. 2023 Aug 21;14:211. doi: 10.1186/s13287-023-03432-2 (PMC10441722; doi:10.1186/s13287-023-03432-2)
Supplement: Supplementary file 3 — Additional file 3: Figure S2. hUC-MSC transplantation effects on spleen weight of 14-week-old MRL/lpr mice. Spleen weight ratio of mice in the MSC transplantation groups (MD and HD groups) showed a decrease compared with the Ctrl. (Ctrl: control, LD: low dose, MD: middle dose, HD: high dose). Compare the distance measured by two rulers of different units. The result indicated that the distance measurement was the same. The number of mice is n = 5. [file 13287_2023_3432_MOESM3_ESM.docx]

**Additional file 3: Figure S2**.


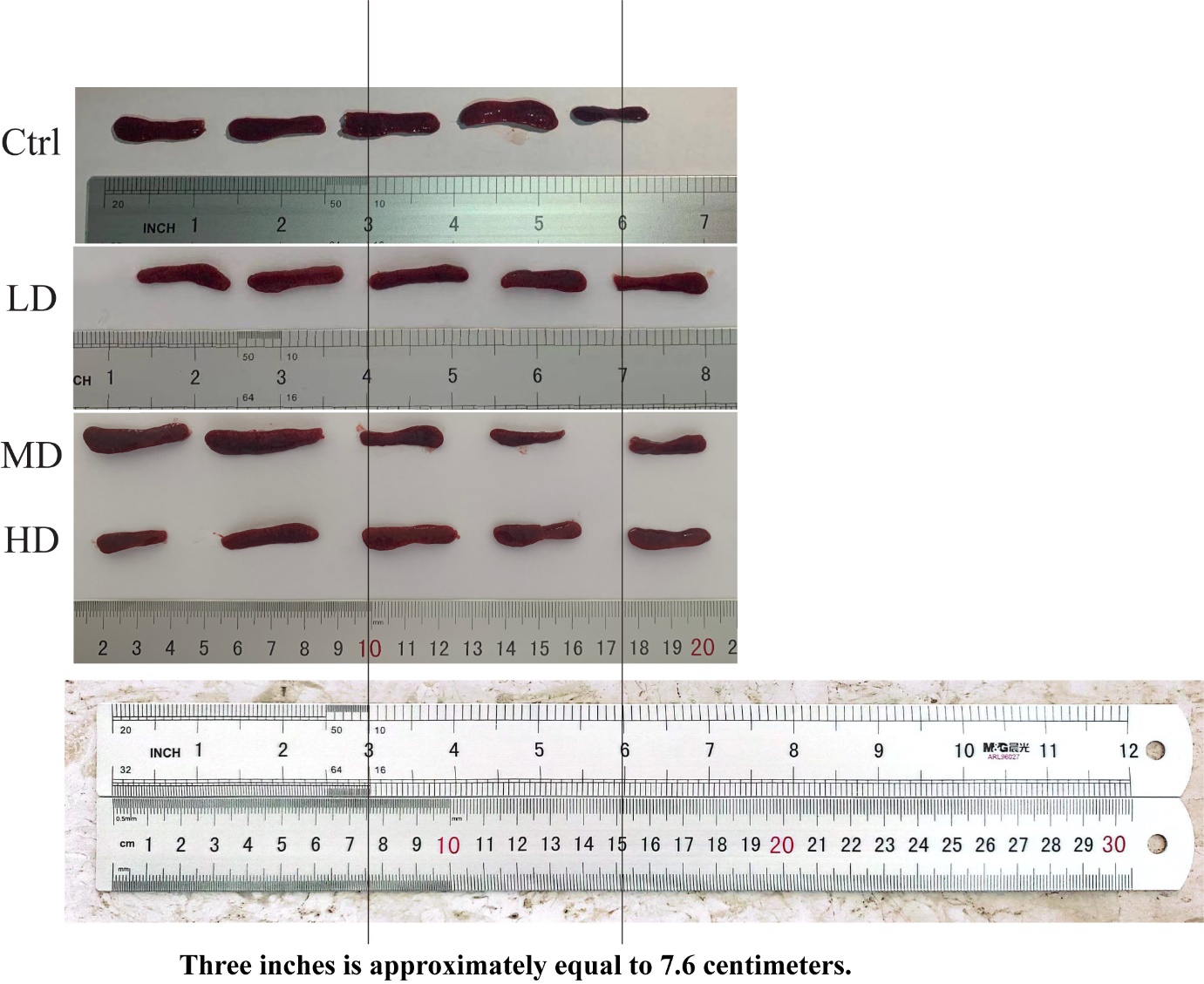


**Additional file 3: Figure S2**. hUC-MSC transplantation effects on spleen weight of 14-week-old MRL/lpr mice. Spleen weight ratio of mice in the MSC transplantation groups (MD and HD groups) showed a decrease compared with the Ctrl. (Ctrl: control, LD: low dose, MD: middle dose, HD: high dose). Compare the distance measured by two rulers of different units. The result indicated that the distance measurement was the same. The number of mice is n = 5.
